# High frequency, cell type-specific visualization of fluorescent-tagged genomic sites in interphase and mitotic cells of living *Arabidopsis *plants

**DOI:** 10.1186/1746-4811-6-2

**Published:** 2010-01-19

**Authors:** Antonius JM Matzke, Koichi Watanabe, Johannes van der Winden, Ulf Naumann, Marjori Matzke

**Affiliations:** 1 Gregor Mendel Institute of Molecular Plant Biology, Austrian Academy of Sciences, Dr. Bohr-Gasse 3, A-1030 Vienna, Austria; 2 Leibniz-Institut für Pflanzengenetik und Kulturpflanzenforschung (IPK), Correnstrasse 3, D-O6466 Gatersleben, Germany

## Abstract

**Background:**

Interphase chromosome organization and dynamics can be studied in living cells using fluorescent tagging techniques that exploit bacterial operator/repressor systems and auto-fluorescent proteins. A nuclear-localized Repressor Protein-Fluorescent Protein (RP-FP) fusion protein binds to operator repeats integrated as transgene arrays at defined locations in the genome. Under a fluorescence microscope, the tagged sites appear as bright fluorescent dots in living cells. This technique has been used successfully in plants, but is often hampered by low expression of genes encoding RP-FP fusion proteins, perhaps owing to one or more gene silencing mechanisms that are prevalent in plant cells.

**Results:**

We used two approaches to overcome this problem. First, we tested mutations in four factors involved in different types of gene silencing and/or epigenetic modifications for their effects on nuclear fluorescence. Only mutations in DDM1, a chromatin remodelling ATPase involved in repeat-induced heterochromatin formation and DNA methylation, released silencing of the RP-FP fusion protein. This result suggested that the operator repeats can trigger silencing of the adjacent gene encoding the RP-FP fusion protein. In the second approach, we transformed the tagged lines with a second T-DNA encoding the RP-FP fusion protein but lacking operator repeats. This strategy avoided operator repeat-induced gene silencing and increased the number of interphase nuclei displaying fluorescent dots. In a further extension of the technique, we show that green fluorescent-tagged sites can be visualized on moving mitotic chromosomes stained with red fluorescent-labelled histone H2B.

**Conclusions:**

The results illustrate the propensity of operator repeat arrays to form heterochromatin that can silence the neighbouring gene encoding the RP-FP fusion protein. Supplying the RP-FP fusion protein in *trans *from a second T-DNA largely alleviates this problem. Depending on the promoter used to drive expression of the RP-FP fusion protein gene, the fluorescent tagged sites can be visualized at high frequency in different cell types. The ability to observe fluorescent dots on both interphase and mitotic chromosomes allows tagged sites to be tracked throughout the cell cycle. These improvements enhance the versatility of the fluorescent tagging technique for future studies of chromosome arrangement and dynamics in living plants.

## Background

Interphase chromosome arrangement and dynamics are increasingly recognized as factors that contribute to the regulation of eukaryotic gene expression [1-6]. The study of interphase chromosomes has been enhanced by techniques for marking genomic sites with fluorescent tags, which allow visualization and tracking of specific regions of the genome in living, untreated cells [7]. The technique is based on bacterial operator repressor systems such as the Lac repressor-*lac *operator (LacI-*lacO*) and the Tet repressor-*tet *operator (TetR-*tetO*) [8]. A nuclear-localized translational fusion between the repressor protein (RP) and an auto-fluorescent protein (FP) recognizes and binds to operator repeats that are integrated as a transgene array into the genome. The tagged sites can be viewed as bright fluorescent dots under a fluorescence microscope.

Fluorescence tagging has been used successfully to investigate interphase chromosome organization and dynamics in living plant cells [8-10]. However, weak and non-uniform nuclear fluorescence, possibly due to silencing of genes encoding RP-FP fusion proteins, often hinders the use of this technology in plants. The problem is particularly pronounced in root cells, which are otherwise well suited for this technique because of their low background fluorescence at commonly used excitation wavelengths [8,11]. Here we describe strategies to improve the expression of the RP-FP fusion protein and the consistency of the fluorescent signals in interphase nuclei of different cell types. In addition, we demonstrate that the fluorescent tagged sites can be visualized and tracked on mitotic chromosomes in living cells.

## Results and Discussion

### Effects of mutations in epigenetic factors on expression of the RP-FP fusion protein

**Figure 1:**
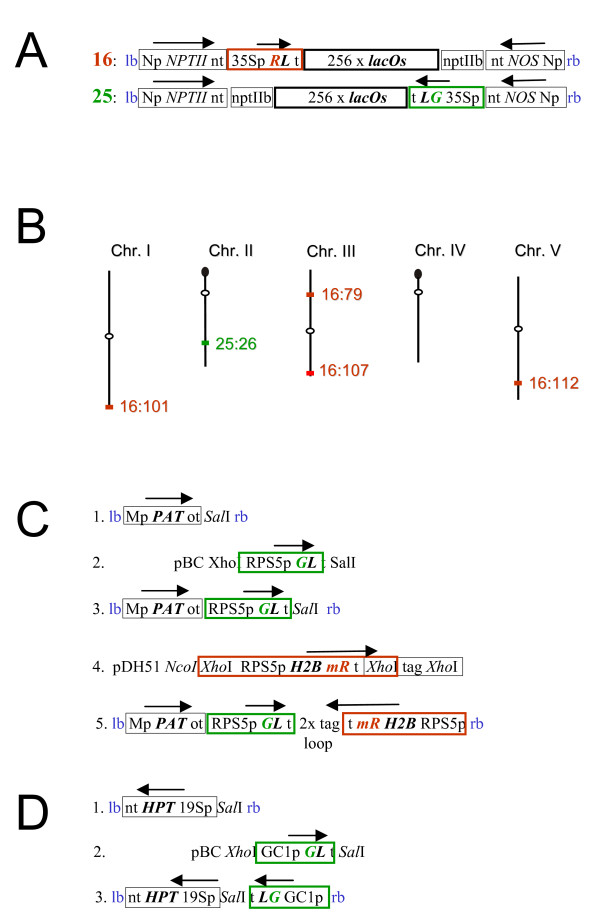
**Constructs and chromosomal locations of fluorescent-tagged sites**. A. T-DNA constructs for fluorescent tagging. Construct 16 is based on red fluorescent protein (R). Construct 25 is based on enhanced green fluorescence protein (G). Both constructs use the *lacO*s and the LacI (L) fused to R and G. Genes encoding RL and GL are under the control of the 35S promoter of CaMV (35Sp). B. Chromosomal positions of tagged sites. The insertion sites have been reported previously [8,11]. Lines are named according to the construct used (16 or 25) followed by the line number (26, 79, 101, 107, 112). C. Constructs containing the RPS5 promoter to drive expression of genes encoding the EGFP-LacI fusion protein (*GL*) and histone H2B fused to monomeric DsRed (*H2BmR*). D. Constructs containing the GC1 promoter to drive expression of *GL*. Details of construct assembly using the modules shown in C and D are in the Methods section. Gene units are boxed; heavy outlines indicate genes encoding fluorescent fusion proteins (red, green) and *lacO*s (black). Arrows indicate the directions of transcription. Additional abbreviations: lb, T-DNA left border; rb, T-DNA right border; Np, NOS promoter; red 'R', DsRed2; green 'G', EGFP; t, transcriptional terminator from the 35S transcript of CaMV; nptIIb, neomycin phosphotransferase II for selection of bacteria on kanamycin; nt, NOS terminator; Mp, mannopine synthase promoter; ot, octopine synthase terminator; tag, 300 bp filler sequence; 19Sp, 19S promoter of CaMV; pDH51, pUC18 containing the 35S promoter-35S terminator cassette [33]; pBC, Bluescript plasmid encoding chloramphenicol resistance.

For the tagged lines developed in our laboratory, a possible reason for low nuclear fluorescence is silencing of the gene encoding the RP-FP fusion protein, which is adjacent to the operator repeats and under the control of the 35S promoter on the original T-DNA constructs (Fig. 1A). To test the occurrence and basis of epigenetic silencing, we introgressed mutations in four epigenetic silencing factors into five tagged lines (Fig. 1B). The epigenetic factors were chosen because of their involvement in different types of gene silencing and/or epigenetic modifications: (1) DDM1 is a chromatin remodelling ATPase important for repeat-induced heterochromatin formation and DNA methylation [12]; (2) DRD1 is a chromatin remodelling ATPase required for RNA-directed DNA methylation and transcriptional gene silencing (TGS) [12]; (3) RDR6 is an RNA-dependent RNA polymerase required for post-transcriptional gene silencing (PTGS), which is often observed with 35S promoter-driven transgenes [13]; (4) MOM1 is an unusual putative chromatin remodelling factor needed for TGS that is independent of DNA methylation [14].

**Figure 2:**
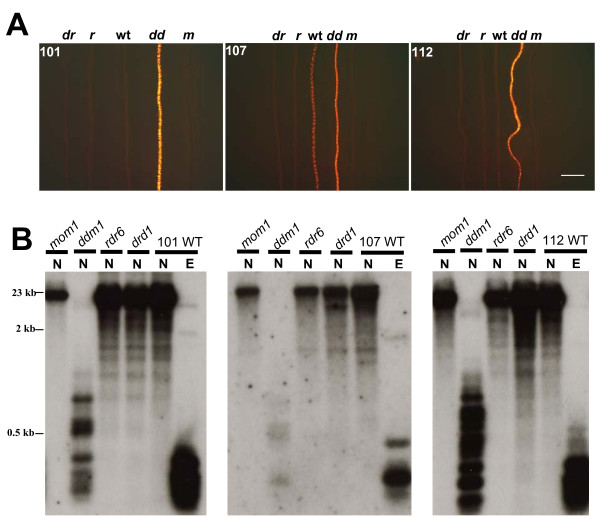
**Release of silencing of the *DsRed-LacI *gene and loss of DNA methylation from *lac *operator repeats in a *ddm1 *mutant**. A. DsRed fluorescence in roots of seedlings doubly homozygous for a tagged locus (101, 107, 112) and an epigenetic mutation: *drd1 *(*dr*), *rdr6 *(*r*), wild type (wt), *ddm1 *(*dd*), *mom1 *(*m*). Increased fluorescence is observed only in the *ddm1 *mutant. The bar indicates 2 mm. B. DNA methylation analysis of *lac *operator repeats. The *lac *operator repeat array is ~9.2 kb (256 copies of a 24-bp *lac*O monomer plus a 12-bp linker sequence) [8]. The *lac *operator repeat arrays can potentially be cut into 315 bp fragments by the restriction enzymes *Eco*RI (E) and *Nar*I (N) [15]. Whereas the former is insensitive to cytosine methylation, *Nar*I will not cleave if the CG in its recognition site (GGCGCC) is methylated (compare N versus E lanes in the three WT panels). Retention of the higher band (*mom1, rdr6 *and *drd1 *lanes) after *Nar*I digestion signifies wild type levels of CG methylation; production of a ladder of smaller bands (*ddm1 *lanes) indicates loss of CG methylation. Size markers are indicated to the left. Data are shown for three tagged lines (101, 107, 112) into which we successfully introduced all four mutations (*drd1*, *ddm1*, *sgs2*, *mom1*). For the other two tagged lines (26, 79), we were unable to generate homozygous mutants for all four of the epigenetic factors. However, we did successfully introgress the *ddm1 *mutation into these lines, which resulted in loss of CG methylation from the *lac *operator repeats in *ddm1 *(Additional file 1).

Plants that are homozygous for the respective mutations were crossed with the homozygous tagged lines and the resulting F1 progeny were allowed to self-fertilize to produce a segregating F2 population. F2 seedlings were genotyped to identify ones that are doubly homozygous for the tagged locus and the mutation in the desired epigenetic factor. Doubly homozygous F2 seedlings were screened for fluorescence in root cells at low magnification. Of the four mutations tested, only *ddm1 *improved expression of the RP-FP fusion protein, which was easily visualized under a fluorescence microscope in extended roots of doubly homozygous F2 seedlings (Fig. 2A, Additional file 1). The release of silencing in the *ddm1 *mutant was accompanied by a substantial loss of DNA methylation from the operator repeats (Fig. 2B, Additional file 1). No improvement of fluorescence or loss of methylation was observed in *rdr6, drd1 *or *mom1 *mutants (Fig. 2A, B; Additional file 1).

Our findings indicate that silencing of the gene encoding the RP-FP fusion protein is correlated with DDM1-dependent DNA methylation of the adjacent operator repeats (Fig. 1A). However, expression of the RP-FP fusion protein is so high in the *ddm1 *mutant that nuclei are filled with fluorescence and individual dots are not discernable (data not shown). Therefore, it is not feasible to perform experiments on interphase arrangement and dynamics of tagged loci in a *ddm1 *mutant background.

In an independent study with another tagged line, named EL702C, *ddm1 *and another DNA hypomethylation mutant, *met1*, were also found to decrease methylation of *lac *operator repeats. However, loss of methylation did not alter the expression of operator repeat-associated genes, including the gene encoding the RP-FP fusion protein (*GFP-LacI*) [15]. Line EL702C may be similar to our line 26, which is exceptional in that methylation is lost from the operator repeats in the *ddm1 *mutant background but the expression of the RP-FP fusion protein, which is already quite high in wild type plants, does not increase (Additional file 1). It is not yet known why the gene encoding the RP-FP fusion protein is protected from silencing in line 26. One possibility is that there is a copy of the gene that is separated from the operator repeats owing to rearrangements within the integrated T-DNA copies. Operator repeats have been used in transgene constructs designed for chromatin charting of epigenetic regulators and gene expression at the T-DNA insertion site [16,17]. These constructs may not always be reliable indicators of expression at the insertion site if operator repeats can potentially form methylated chromatin that induces silencing of adjacent genes.

### Supplying the RP-FP fusion protein in trans: Use of cell type-specific promoters

**Figure 3:**
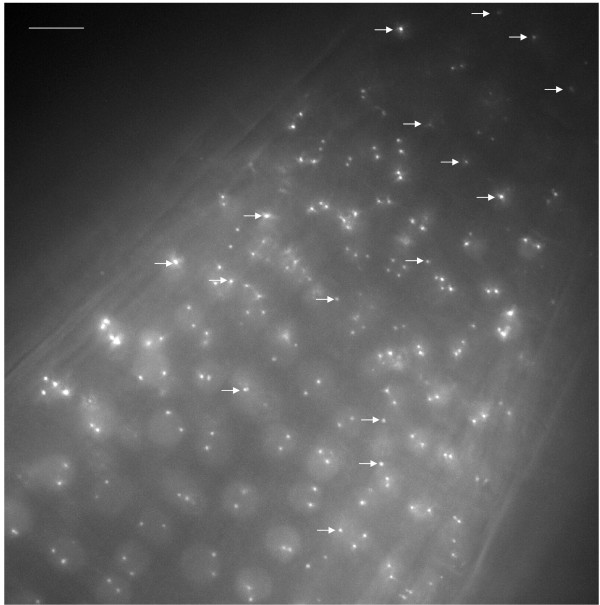
**Fluorescent dots in nuclei of cells in root division zone**. High magnification image of the division zone from a root of a seedling homozygous for the 112 tagged locus (Fig. 1B). The gene encoding the EGFP-LacI fusion protein is under the control of the RPS5 promoter, which is active in this region of roots. In the majority of nuclei, two separate dotes are visible. In about 15%, however, only a single dot is resolved (arrows). For each nucleus, the 3D inter-allelic distance in micrometers was determined using Imaris software for point detection and point-to-point distance measurements (Methods section). Values are shown graphically in Figure 5. The bar indicates 10 μm. The image shows a maximum projection in which all 41 optical sections are collapsed into one plane.

**Figure 4:**
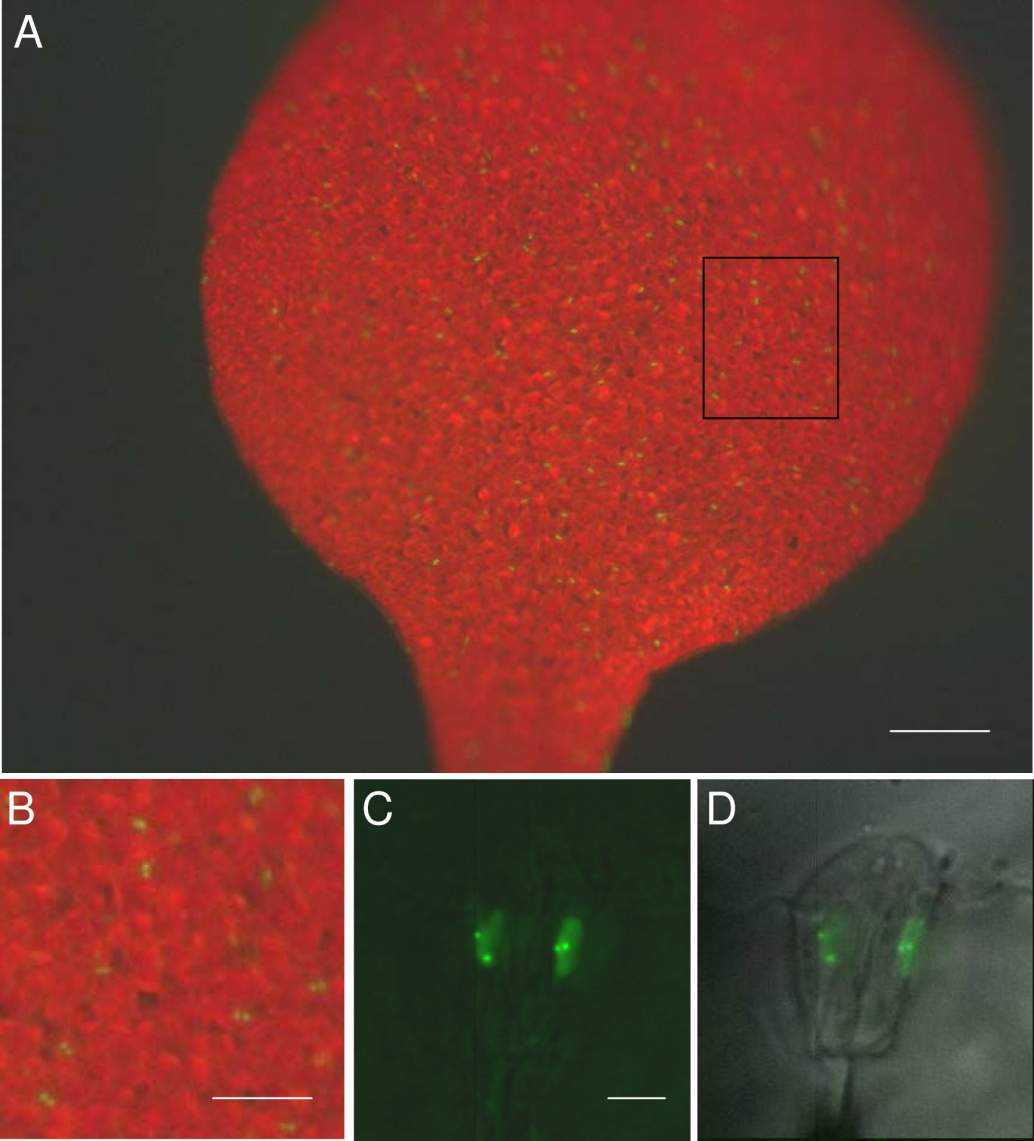
**Fluorescent dots in nuclei of guard cells**. Images from a leaf of a plant homozygous for the 112 tagged locus. The gene encoding the EGFP-LacI fusion protein is under the control of the GC1 (guard cell) promoter. In the low magnification images (A, B), the GFP2 filter was used (at the excitation wavelength for GFP, chlorophyll has red auto-fluorescence using this filter). Many paired green dots, corresponding to guard cell nuclei, are visible in A (enlargement of boxed region shown in B). In the high magnification images (C, D), a YFP filter was used to see fluorescent dots (this reduces chlorophyll auto-fluorescence); D is the superimposition of the light microscopic image. Two fluorescent dots corresponding to the tagged sites are clearly visible above low background fluorescence in both diploid guard cell nuclei. The bars indicate 250 μm (A), 100 μm (B), and 5 μm (C and D).

The correlation between loss of methylation from the operator repeats and derepression of the adjacent gene encoding the RP-FP fusion protein in the *ddm1 *mutant suggested that silencing is a local, *cis*-acting effect. This suggestion is substantiated by the failure of the *rdr6 *mutation to release silencing of the RP-FP fusion protein, which indicates the absence of diffusible, *trans*-acting siRNAs capable of triggering PTGS. We thus tried a second strategy to improve the fluorescence-tagging technique by introducing a second T-DNA that encodes the RP-FP fusion protein but lacks operator repeats. This step also permits the replacement of the 35S promoter, which was used to drive expression of the gene encoding the RP-FP fusion protein in the original constructs that contain operator repeats (Fig. 1A), with cell type-specific promoters. The efficacy of this strategy is illustrated here for two different promoters: the RPS5 promoter (Fig. 1C), which is active in cells of the division zone of the root tip [18,19], and the pGC1 promoter (Fig. 1D), which is active in guard cells [20]. With each promoter, we observed a high frequency of nuclei containing fluorescent dots in the respective cell type (root cells, Fig. 3; guard cells, Fig. 4). By contrast, when the 35S promoter is used to transcribe the gene encoding the RP-FP fusion protein, expression is highly variable and occurs mainly in a subset of cells in the upper part of the root, not in the division zone, and sporadically in leaves and in ovules [21].

**Figure 5:**
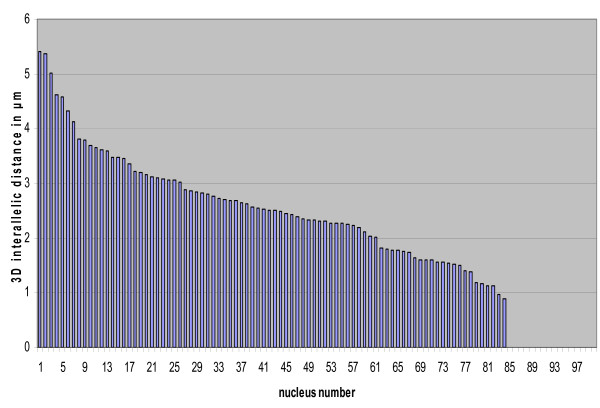
**Range of inter-allelic distances in cells of root division zone**. 3D inter-allelic distances in interphase nuclei of the root division zone (shown in Fig. 3) were measured using Imaris software as described in the Method section. The values were entered into Excel and the graph was produced by sorting in descending order. For nuclei numbered 86 to 100, two distinct dots were not distinguished by Imaris software. These are displayed on this graph as 'zero' and indicated by white arrows in Fig. 3. Possible explanations for this are discussed in the text.

High frequency and uniform expression of the RP-FP fusion protein through the use of cell type-specific promoters allows the measurement of significant numbers inter-allelic 3D distances in neighbouring cells of homozygous tagged lines. In cells of the root division zone, inter-allelic distances comprise a continuum of values, with most ranging between 1 μm to 5 μm (Fig. 5). The rather wide range of inter-allelic distances is consistent with previous results on smaller sample sizes from other cell types and suggests an essentially random arrangement of interphase chromosomes [11,22].

If the inter-allelic distance is less than 1 μm, two distinct dots can be difficult to distinguish. This was observed in a fraction of the nuclei in the root division zone (Fig. 3, arrows), suggesting the inter-allelic 3D distance is less than 1 μm in these cells (Fig. 5). While these might represent cases of somatic pairing arising from associations between operator repeat arrays [15,23,24], other explanations are also possible. For example, one dot of a pair may be located outside of the imaged stack. Another possibility is that dots may be in close proximity if the tagged sites cluster at the metaphase plate in mitotic cells (see below). In an independent study, homologous pairing was reported to be higher for the *lac *operator arrays than for neighbouring euchromatic regions and was only partially reduced in the DNA hypomethylation mutants *ddm1 *and *met1 *[15]. This finding suggested that methylated operator repeats tend to pair in a manner that perturbs the natural spatial arrangements of interphase chromosomes [23]. As described above, we were unable to assess the influence of the *ddm1 *mutation and hypomethylation of the operator repeats on interallelic associations in our study because of the extremely high background fluorescence in *ddm1 *mutant. In general, however, we observe two separate dots in the majority of homozygous nuclei in the cell types tested (Figs. 3 and 4). A recent fluorescence in situ hybridization study found that the frequencies of homologous pairing of transgene repeat arrays may differ with the construct, chromosomal integration site, cell type, and number and repetitiveness of inserts [24].

### Visualizing fluorescent-tagged sites on mitotic chromosomes

Fluorescent-tagging of genomic sites has been useful for studying interphase chromosome dynamics ('single particle tracking') in living yeast and animal cells [25,26]. These studies have shown that chromatin generally diffuses through a restricted volume of the interphase nucleus but can undergo rapid directed movement in an energy-dependent mechanism over brief periods [26]. Single particle tracking in plants has also indicated constrained movement of tagged sites over short time periods [10,11,27]. Whether energy-dependent directed movement is also observed in plant cells in response to developmental or environmental cues remains to be determined.

**Figure 6:**
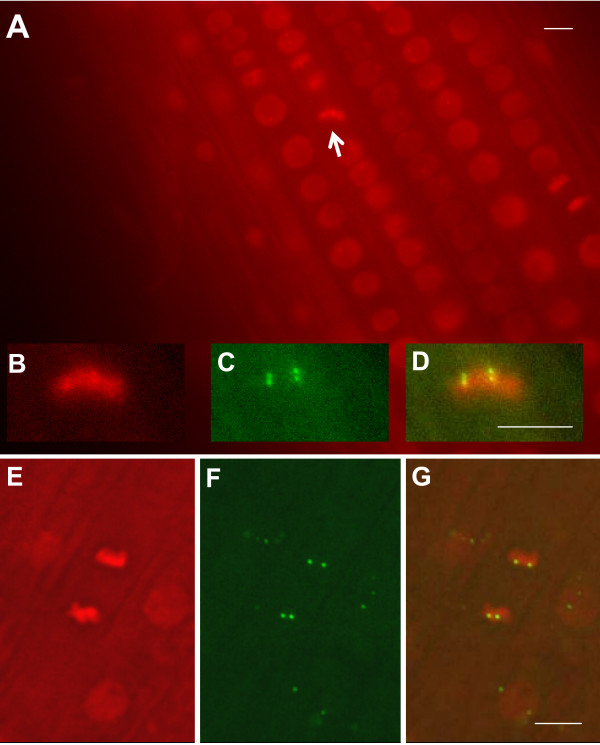
**Visualization of fluorescent tagged sites on mitotic chromosomes**. In the division zone of the root, chromosomes are dual labelled with H2B-mRed and EGFP-LacI (both genes are under control of the RPS5 promoter). (A) Mitotic chromosomes at the metaphase plate are viewed as bright red bars (white arrow; shown enlarged in B) with EGFP-tagged sites visible as two clustered pairs of green fluorescent dots (C; merged image in D). (E) Mitotic chromosomes at anaphase with two pairs of EGFP-tagged sites (F; merged image in G). Interphase nuclei are dull red ovals (A, E, G). In the merged image (G), three neighbouring interphase nuclei with superimposed green fluorescent dots can be seen. All bars indicate 5 μm. Homozygous tagged line 112 was used for A-D; homozygous tagged line 26 was used for E-G. In both cases, the EGFP-LacI fusion protein was supplied in trans.

Whereas fluorescence-tagged sites appear to be rather static in plant interphase nuclei, the situation is different in mitotic cells. Observations of the fluorescence-tagged sites in these cells should be possible provided the RP-FP fusion protein can bind to operator repeats on highly condensed mitotic chromosomes. To test this possibility, we introduced into the tagged lines a second transgene construct encoding EGFP-LacI fusion protein and histone H2B fused with red fluorescent protein (Fig. 1C, construct 5), which uniformly stains *Arabidopsis *chromatin [28]. When the RPS5 promoter is used to drive high frequency expression of both of these genes in cells of the root division zone, green fluorescent dots can be seen superimposed on red fluorescent mitotic chromosomes (Fig. 6A-G). Dual fluorescence labelling allows tracking of tagged sites on mitotic chromosomes as they separate and decondense in daughter cells (Additional files, 2, 3, 4). In principle, it should be possible to follow complete mitotic cycles and analyze whether fluorescent tagged sites are repositioned in daughter cell nuclei, a process that may provide opportunities for alterations in gene expression [1].

## Conclusions

We have tested successfully several strategies for improving the chromosome fluorescence tagging technique in plants. Supplying the RP-FP fusion protein in *trans *avoids the problem of gene silencing associated with the operator repeats, which are targets of DDM1-dependent DNA methylation. Despite heavy methylation of the operator repeats, the RP-FP fusion protein is able to recognize and bind to these sequences in interphase nuclei. Moreover, the RP-FP fusion protein can also bind to operator repeats on highly condensed mitotic chromosomes. This robust and penetrant binding will allow the spatial organization and dynamics of tagged sites to be studied during the cell cycle and may provide a model for epigenetic inheritance of chromosomal proteins through mitosis [29]. Optimal visualization of fluorescent-tagged sites requires a suitable promoter to drive expression of the gene encoding the RP-FP fusion protein. Expression should be uniform within an organ or cell type and should not be too high, otherwise fluorescent dots will be obscured by high background fluorescence. Whereas the 35S promoter does not always fulfil these criteria, cell type-specific promoters often provide the desired level and consistency of expression. We see little evidence for frequent pairing of fluorescent tagged alleles in interphase nuclei despite high levels of DNA methylation at the operator repeats. Thus, fluorescent tagged sites should generally provide accurate indicators of natural spatial relationships of interphase and mitotic chromosomes in living cells. The improvements we have described will allow the fluorescence tagging technology to be more fully exploited in plants to study chromosome organization and dynamics during growth and development and under various environmental conditions.

## Methods

### Plant material

The fluorescence tagged lines (*Arabidopsis thaliana *ecotype Col-0) used in this study were described in previous publications [8,11]. Transformation of *A. thaliana *was carried out using the floral dip method [30]. Transformed seedlings were selected on solid Murashige and Skoog (MS) medium containing 500 mg/l claforan and 20 mg/l phosphinotricin (Fig. 1C, constructs 1, 3 and 5) or 20 mg/l hygromycin (Fig. 1D, constructs 1 and 3).

### Construction of recombinant plasmids

#### Binary vectors

BV-Mp*PAT*ot *Sal*I (Fig. 1C, construct 1) is a binary vector modified to contain a *Sal*I site [31]. BV-nt*HYG*19Sp *Sal*I (Fig. 1D, construct 1) is a binary vector modified to contain a *Sal*I site [32]. These vectors allow selection of transformed plants on phosphinotricin and hygromycin, respectively.

#### RP-FP constructs under the control of the RPS5 or GC1 promoters

The RPS5 promoter (At3g11940) [18] was isolated in two fragments by PCR from genomic DNA of *A. thaliana *ecotype Col-0: (1) *Xho*I-420-*Eco*RI and (2) *Xho*I-*Eco*RI-1230-*Nhe*I. The *Xho*I 420-*Eco*RI fragment was amplified using the following primers and cloned into pBC (Stratagene, La Jolla, USA):

5'-GCG CTC GAG CCC ATA ATC GTG AGT AGA TAT ATT ACT CAA C-3'

5'-CGC GAA TTC ACC TGA GGC ATG TAG AAA GCT AGT GAT ATG G-3'

The *Xho*I-*Eco*RI-1230-*Nhe*I fragment was amplified using the following primers and cloned into pBluescript (Stratagene, La Jolla, USA) modified to contain an additional *Nhe*I site:

5'-GCG CTC GAG CTA GAA TTC ATC TAT TTC CGT CTT AAC TAT TTC-3'

5'-CGC GCT AGC GAG CTC AAA TAC ACA AGT ACG AAA TCA GAG AGG-3'.

These fragments were combined by inserting the *Xho*I- 420-*Eco*RI fragment between the *Xho*I- *Eco*RI- sites of the *Xho*I-*Eco*RI- 1230 - *Nhe*I fragment in Bluescript (Stratagene, La Jolla).

To obtain the GC1 promoter (At1g22690) [20], a *Xho*I GC1p- *Nhe*I fragment was synthesized by Mr.Gene (Regensburg, Germany). The -1140/+23 fragment was chosen because it was reported to give the strongest expression levels [20].

To make the final constructs, pBC-*Xho*I-35Sp *Nhe*I -*GL *t -*Sal*I (see Fig. 16.4, part b in reference [8]) was cut with *Xho*I and *Nhe*I releasing the 35Sp. The 35Sp was replaced with a fragment containing the RPS5 promoter (*Xho*I RPS5p *Nhe*I) leading to the construct *Xho*I RPS55 *Nhe*I - *GL *t- *Sal*I (Fig. 1C, construct 2) or with a fragment containing the GC1 promoter (*Xho*I GC1p *Nhe*I) leading to *Xho*I GC1p- *Nhe*I -*GL *t *Sal*I (Fig. 1D, construct 2). Then these constructs were cut with *Xho*I and *Sal*I and ligated into the *Sal*I-cut binary vector BV-Mp*PAT*ot-*Sal*I (Fig. 1C, construct 1) or *Sal*I-cut binary vector BV-nt*HYG*19Sp-*Sal*I (Fig. 1D, construct 1), respectively, leading to the constructs shown in Fig. 1C, construct 3 and Fig. 1D, construct 3. The construct shown in Fig. 1C, construct 5 has been introduced into all five tagged lines (Fig. 1B); the construct shown in Fig. 1D, construct 3 has only been introduced into line 112.

#### H2B constructs

A construct encoding a histone H2B-DsRed monomer fusion protein under the control of the RPS5 promoter (Fig. 1C, construct 4: pDH51 *Nco*I *Xho*I RPS5 *Nhe*I *Bam*HI - *H2B *- *Bam*HI *mRed Xba*I t -*Hin*dIII *Xho*I tag *Xho*I *Hin*dIII) was produced as follows:

First, the *Xho*I-RPS5-NheI promoter fragment (as described above in RP-FP constructs section) was adjusted for insertion into a modified pDH51 (see below) to a *Nco*I-*Xho*I-RPS5-*Nhe*I-*Bam*HI fragment by adding a *Xho*I-*Nco*I-tag-*Nco*I-*Xho*I tag into the *Xho*I site add a *Nhe*I-*Bam*HI tag-*Bam*HI-*Nhe*I into the *Nhe*I site.

pDH51 [33], which contains *Nco*I-35S promoter-*Bam*HI-*Xba*I-35S terminator *Hin*dIII (only restriction enzymes necessary for the construct are mentioned), was modified by inserting *Xho*I tags into the *Nco*I and *Hin*dIII sites of this plasmid. A gene encoding DsRed-monomer (Clontech, Palo Alto, USA) was inserted as *Bam*HI- *Xba*I fragment. Then the *Nco*I-35S promoter-*Bam*HI fragment was replaced by the NcoI-XhoI-RPS5-NheI-BamHI fragment described above. Finally, a fragment containing the gene encoding histone H2B (At5g22880) [28], *Bam*HI - *H2B *- *Bam*HI, was isolated by PCR from genomic DNA of *A. thaliana *ecotype Col-0 using the following primers:

5'-CGC GGA TCC ATG GCG AAG GCA GAT AAG AAA CCA GCG GAG-3'

5'-CGC GGA TCC CCA GAA CTC GTA AAC TTC GTA ACC GCC TTA G-3'

This fragment was inserted in the right orientation into the *Bam*HI site before the gene encoding the DsRed-monomer. This construct was released with *Xho*I and cloned into the *Sal*I site of the binary vector shown in Fig. 1C, construct 3, resulting in Fig. 1C, construct 5. The inverted orientation of the 35S terminator regions (t) was stabilized by insertion of two copies of the *Xho*I tag of the construct in Fig. 1C, construct 4. All constructs were confirmed by sequencing.

### Epigenetic mutants

The mutant alleles used in this study are: *mom1-1, drd1-6, rdr6 *(*sgs2-1*) and *ddm1-5 *(*som8*) http://www.epigenome-noe.net/resources/scilinks.php. Mutations were genotyped using the following primers:

*mom1-1*:

CD 34: 5' AAG AGC TGT TAC ACC TGC TGA ATG C 3'

CD 38: 5' CAG TTG TAA CCG GTG GAT CTC CTG T 3'

SALK LBa1: 5' TGG TTC ACG TAG TGG GCC ATC G 3'

Use of CD 34/CD 38 yields a fragment of 486 bp for wild type plants. Use of CD 38/LBa1 yields a fragment of 461 bp for the *mom1 *mutant.

*drd1-6*:

drd1-6 F: 5' AGC TAA GGG ATG GAA ACT AGG 3'

rd1-6 R: 5' CGA GAT GCT CCA ACA AGC GAG 3'

Digestion with *Nde*II yields a 168 bp fragment for wild type and two fragments (73 and 95 bp) for *drd1-6*.

*rdr6/sgs2-1*:

29IIF7: 5' GCA GGG ATA CTT GAA CAT GGC C 3'

29IIR3: 5' CAA ACA TTT GTG ACC CCA TGC C 3'

Digestion with *Bst*NI yield three fragments (150, 250 and 850 bp) for wild type and two fragments (400 and 850 bp) for *rdr6/sgs2-1*.

*ddm1-5*:

F5' AAG CGA CGG AGA CGA CTG TTT G 3'

R5' TTT CAC AAA GCA ACC ACA CTA CG 3'

The fragment produced in the *ddm1-5 *mutant is approximately 0.5 kb; in wild type fragment is approximately 0.35 kb.

In all cases, the following PCR conditions were used: 1 min 95°C; [30 sec 95°C; 30 sec 55°C; 1 min 72°C; 50×]; 6 min 72°. The products were run on a 2.5% agarose gel.

### Epi-fluorescence image acquisition

*A. thaliana *seedlings were germinated and grown on solid MS medium under a 16 hour light-8 hour dark cycle at 23°C. For pre-screening under low magnification for fluorescence in roots and leaves, untreated plantlets growing on solid MS medium in Petri dishes were examined on a Leica MZ16FA, fluorescence stereo microscope (Leica, Wetzlar, Germany) equipped with the following filters: Leica 10447227 (DsRed) and GFP2 Leica (10447221) (Leica, Wetzlar, Germany). Photographs were taken using a DFC300FX colour camera (Leica, Wetzlar, Germany).

For viewing fluorescent dots at high magnification, seedlings were removed from MS medium approximately 10 to 30 days after germination and mounted on a microscope slide in tap water. For roots, a slide with an indentation was used (Assistant, Sondheim, Germany, Cat. Nr. 2410). Leaves were mounted in a Series 20 Chamber (Model RC-26G, Warner Instruments, Hamden, USA) [34]. Roots were viewed using a Zeiss Axioplan 2 fluorescence microscope (Zeiss, Jena, Germany) with a Spot Pursuit charge coupled device (CCD) camera (Visitron, Puchheim, Germany) or a Zeiss Axiovert 200 M with Photometrix CCD camera Quantix (Visitron, Pucheim, Germany) [8]. Leaves were viewed with the latter microscope, which is an inverted microscope that can accommodate the Series 20 chamber.

To make optical sections (stacks) using both microscope set-ups, the multidimensional acquisition tool from Metamorph software (Molecular Devices, MDS Analytical Technologies, Toronto) was used. AutoDeblure (Autoquant, purchased from Bitplane, Zürich) was used for deconvolution. Imaris software Version 6.1.3 was used for point detection and point to point distance measurements (Imaris MATLAB extensions, purchased from Bitplane, Zürich). The dot diameter was set to 0.5 μm. One stack consists of approximately 40 acquisitions each separated by a distance of 0.2 μm. With an exposure time of one second per picture, one stack takes approximately 2 minutes for one color and four minutes with two colors.

### Analysis of DNA methylation

DNA methylation at the *lac *operator repeats was analyzed using a methylation-sensitive restriction enzyme (*Nar*I) and Southern blotting. DNA was isolated from rosette leaves of adult plants as described previously [8]. Digestion with *NarI *and *EcoRI *(methylation-insensitive) was carried out according to the manufacturer's instructions (New England Biolabs, Massachusetts, USA). Protocols for Southern blotting and hybridization as well as the probe used to detect *lac *operator repeats were described previously [8].

## Abbreviations

3D: three dimensional; CaMV: cauliflower mosaic virus; CCD: charge-coupled device; DDM1: decrease in DNA methylation1; DRD1: defective in RNA-directed DNA methylation1; DsRed or just R: *Discosoma *sp. red fluorescent protein; EGFP or just G: enhanced green fluorescent protein; GFP: green fluorescent protein; R: DsRed2; mRed or mR: DsRed-monomer fluorescent protein; GC1p or pGC1: guard cell promoter; H2B: histone H2B; HPT: hygromycin phosphotransferase; Lac: lactose; LacI: Lac repressor protein; *lacO: lac *operator repeats; MET1: methyltransferase1; MOM1: Morpheus molecule1; MS: Murashige and Skoog medium; NLS: nuclear localization signal; NOS: nopaline synthase; NPTII: neomycin phosphotransferase II; PAT: phosphinotricin-acetyl transferase; PTGS: post-transcriptional gene silencing; RdDM: RNA-directed DNA methylation; RDR6: RNA-dependent RNA polymerase 6; RP-FP fusion protein: repressor protein-fluorescent protein fusion protein; RPS5p or pRPS5: ribosomal protein small subunit 5 promoter; siRNAs: small interfering RNAs; T-DNA: transferred DNA; Tet: tetracycline; TGS: transcriptional gene silencing

## Competing interests

The authors declare that they have no competing interests.

## Authors' contributions

AJMM and MM designed and supervised the study. AJMM, KW, JvdW and UN performed the experimental work. AJMM and MM wrote the paper. All authors read and approved the manuscript.

## Supplementary Material

Additional file 1 **Release of silencing of the RP-FP fusion protein and loss of DNA methylation from *lac *operator repeats in a *ddm1 *mutant**. For two lines, 26 and 79, we were not able to successfully introgress all four epigenetic mutations (*rdr6 *for line 26, and for *mom1 *and *drd1*-6 for line 79). For both lines, however, we did successfully introgress the *ddm1 *mutation, which released silencing of the RP-FP fusion protein and reduced DNA methylation of the operator repeats in lines 101, 107, 112 (Fig. 2A, B). The bars indicate 2 mm. (A) Line 79 displayed strong derepression of the *DsRed-LacI *gene in a *ddm1 *mutant background (right). Line 26 was exceptional in that the *EGFP-LacI *gene was not strongly silenced in wild type plants and no improved expression was observed in the *ddm1 *mutant (left). (B) In both 79 and 26 lines, the operator repeats lost methylation. Click here for file

Additional file 2 **Tracking of fluorescent tagged sites on mitotic chromosomes**. A 3D stack with 33 planes at intervals of 0.2 μm was taken over a period of approximately 4 minutes starting on a metaphase plate in homozygous line 107. Chromosome movement commenced at the beginning of the 4 minute period. Four still shots from a movie (Additional file 3: Movie Metamorph) are shown at the top. Left to right: plane 4, 10, 18, 24. The optical sections can be analyzed in ImarisTrack (Bitplane, Zürich) by changing the Z stack into a time stack and then the moving dots can be tracked and displayed in a rendered computer simulation (Additional file 4: Movie Imaris). Representative frames are shown at the bottom. Click here for file

Additional file 3 **Movie Metamorph**. Movie of fluorescent tagged sites on mitotic chromosomes. Click here for file

Additional file 4 **Movie Imaris**. Rendered computer simulation of fluorescent tagged sites on mitotic chromosomes. Click here for file

## References

[B1] Dillon N The impact of gene location in the nucleus on transcriptional regulation Dev Cell 2008 15 182 186 10.1016/j.devcel.2008.07.013 18694558

[B2] Zhao R Bodnar MS Spector DL Nuclear neighborhoods and gene expression Curr Opin Genet Devel 2009 19 172 179 10.1016/j.gde.2009.02.007 PMC2677118 19339170

[B3] Chuang CH Belmont AS Moving chromatin within the interphase nucleus - controlled transitions? Sem Cell Dev Biol 2007 18 698 796 10.1016/j.semcdb.2007.08.012 PMC2117624 17905613

[B4] Kumaran RI Thakar R Spector DL Chromatin dynamics and gene positioning Cell 2008 132 929 934 10.1016/j.cell.2008.03.004 18358806 PMC2898133

[B5] Takizawa T Meaburn KJ Misteli T The meaning of gene positioning Cell 2008 135 9 13 10.1016/j.cell.2008.09.026 18854147 PMC3478881

[B6] Deniaud E Bickmore WA Transcription and the nuclear periphery: edge of darkness? Curr Opin Genet Dev 2009 19 187 191 10.1016/j.gde.2009.01.005 19231154

[B7] Robinett CC Straight A Li G Willhelm C Sudlow G Murray A Belmont AS In vivo localization of DNA sequences and visualization of large-scale chromatin organization using lac operator/repressor recognition J Cell Biol 1996 135 1685 1700 10.1083/jcb.135.6.1685 8991083 PMC2133976

[B8] Matzke AJM Huettel B Winden J van der Matzke M Fluorescent transgenes to study interphase chromosomes in living plants Methods Mol Biol 2008 463 241 265 full_text 18951172 10.1007/978-1-59745-406-3_16

[B9] Lam E Luo C Watanabe N Charting functional and physical properties of chromatin in living cells Curr Opin Genet Devel 2009 19 135 141 10.1016/j.gde.2009.02.004 19327981

[B10] Luo C Lam E Chromatin charting: global mapping of epigenetic effects Methods Mol Biol 2009 553 127 39 full_text 19588104 10.1007/978-1-60327-563-7_7

[B11] Matzke AJ Huettel B van der Winden J Matzke M Use of two-color fluorescence-tagged transgenes to study interphase chromosomes in living plants Plant Physiol 2005 139 1586 1596 10.1104/pp.105.071068 16339805 PMC1310544

[B12] Matzke M Kanno T Daxinger L Huettel B Matzke AJM RNA-mediated chromatin-based silencing in plants Curr Opin Cell Biol 2009 21 367 376 10.1016/j.ceb.2009.01.025 19243928

[B13] Butaye KM Goderis IJ Wouters PF Pues JM Delauré SL Broekaert WF Depicker A Cammue BP De Bolle MF Stable high-level transgene expression in Arabidopsis thaliana using gene silencing mutants and matrix attachment regions Plant J 2004 39 440 449 10.1111/j.1365-313X.2004.02144.x 15255872

[B14] Caikovski M Yokthongwattana C Habu Y Nishimura T Mathieu O Paszkowski J Divergent evolution of CHD3 proteins results in MOM1 refining epigenetic control in vascular plants PLoS Genet 2008 22 e1000165 10.1371/journal.pgen.1000165 PMC2507757 18725928

[B15] Watanabe K Pecinka A Meister A Schubert I Lam E DNA hypomethylation reduces homologous pairing of inserted tandem repeat arrays in somatic nuclei of *Arabidopsis thaliana* Plant J 2005 44 531 540 10.1111/j.1365-313X.2005.02546.x 16262704

[B16] Rosin FM Watanabe N Cacas JL Kato N Arroyo JM Fang Y May B Vaughn M Simorowski J Ramu U McCombie RW Spector DL Martienssen RA Lam E Genome-wide transposon tagging reveals location-dependent effects on transcription and chromatin organization in Arabidopsis Plant J 2008 55 514 525 10.1111/j.1365-313X.2008.03517.x 18410481

[B17] Luo C Durgin BG Watanabe N Lam E Defining the functional network of epigenetic regulators in *Arabidopsis thaliana* Mol Plant 2009 2 661 674 10.1093/mp/ssp017 19825647

[B18] Weijers D Franke-van Dijk M Vencken R-J Quint A Hooykaas P Offringe R An Arabidopsis minute-like phenotype caused by a semi-dominant mutation in a *RIBOSOMAL PROTEIN S5 *gene Development 2001 128 4289 4299 11684664 10.1242/dev.128.21.4289

[B19] Lindhout BI Fransz P Tessadori F Meckel T Hooykaas PJJ Zaal BJ van der Live cell imaging of repetitive DNA sequences via GFP-tagged polydactyl zinc finger proteins Nucl Acids Res 2007 35 e107 10.1093/nar/gkm618 17704126 PMC2018617

[B20] Yang Y Costa A Leonhardt N Siegel RS Schroeder JI Isolation of a strong *Arabidopsis guard *cell promoter and its potential as a research tool Plant Methods 2008 4 6 10.1186/1746-4811-4-6 18284694 PMC2323621

[B21] Matzke AJM Winden J van der Matzke M Tetracycline operator/repressor system to visualize fluorescence-tagged T-DNAs in interphase nuclei of Arabidopsis Plant Mol Biol Rep 2003 21 9 19 10.1007/BF02773392

[B22] Huettel B Kreil DP Matzke M Matzke AJ Effects of aneuploidy on genome structure, expression and interphase organization in *Arabidopsis thaliana* PLoS Genetics 2008 4 e1000226 10.1371/journal.pgen.1000226 18927630 PMC2562519

[B23] Pecinka A Kato N Meister A Probst AV Schubert I Lam E Tandem repetitive transgenes and fluorescent chromatin tags alter local interphase chromosome arrangement in *Arabidopsis thaliana* J Cell Sci 2005 118 3751 3758 10.1242/jcs.02498 16076901

[B24] Jovtchev G Watanabe K Pecinka A Rosin FM Mette MF Lam E Schubert I Size and number of tandem repeat arrays can determine somatic homologous pairing of transgene loci mediated by epigenetic modifications in *Arabidopsis thaliana *nuclei Chromosoma 2008 117 267 276 10.1007/s00412-007-0146-0 18200447

[B25] Chuang CH Carpenter AE Fuchsova B Johnson T de Lanerolle P Belmont AS Long-range directional movement of an interphase chromosome site Curr Biol 2006 16 825 831 10.1016/j.cub.2006.03.059 16631592

[B26] Levi V Gratton E Chromatin dynamics during interphase explored by single-particle tracking Chromosome Res 2008 16 439 449 10.1007/s10577-008-1240-8 18461483 PMC2701671

[B27] Kato N Lam E Chromatin of endoreduplicated pavement cells has greater range of movement than that of diploid guard cells in *Arabidopsis thaliana* J Cell Sci 2003 116 2195 2201 10.1242/jcs.00437 12692151

[B28] Boisnard-Lorig C Colon-Carmona A Bauch M Hodge S Doerner P Bancharel E Dumas C Haseloff J Berger F Dynamic analyses of the expression of the HISTONE::YFP fusion protein in *Arabidopsis *show that syncytial endosperm is divided in mitotic domains Plant Cell 2001 13 495 509 10.1105/tpc.13.3.495 11251092 PMC135513

[B29] Probst AV Dunleavy E Almouzni G Epigenetic inheritance during the cell cycle Nat Rev Mol Cell Biol 2009 10 192 205 10.1038/nrm2640 19234478

[B30] Clough SJ Bent AF Floral dip: a simplified method for Agrobacterium-mediated transformation of *Arabidopsis thaliana* Plant J 1998 16 735 743 10.1046/j.1365-313x.1998.00343.x 10069079

[B31] Aufsatz W Mette MF van der Winden J Matzke M Matzke AJ HDA6, a putative histone deacetylase needed to enhance DNA methylation induced by double stranded RNA EMBO J 2002 21 6832 6841 10.1093/emboj/cdf663 12486004 PMC139084

[B32] Mette MF van der Winden J Matzke M Matzke AJ Production of aberrant promoter transcripts contributes to methylation and silencing of unlinked homologous promoters in trans EMBO J 1999 18 241 248 10.1093/emboj/18.1.241 9878066 PMC1171118

[B33] Pietrzak M Shillito RD Hohn T Potrykus I Expression in plants of two bacterial antibiotic resistance genes after protoplast transformation with a new plant expression vector Nucleic Acids Res 1986 14 5857 5868 10.1093/nar/14.14.5857 3016666 PMC311596

[B34] Shaw SL Imaging the live plant cell Plant J 2006 45 573 598 10.1111/j.1365-313X.2006.02653.x 16441350

