# Supplementary material for: High frequency, cell type-specific visualization of fluorescent-tagged genomic sites in interphase and mitotic cells of living Arabidopsis plants
Source: Plant Methods. 2010 Jan 19;6:2. doi: 10.1186/1746-4811-6-2 (PMC2820019; doi:10.1186/1746-4811-6-2)
Supplement: Additional file 1 — Release of silencing of the RP-FP fusion protein and loss of DNA methylation from lac operator repeats in a ddm1 mutant. For two lines, 26 and 79, we were not able to successfully introgress all four epigenetic mutations (rdr6 for line 26, and for mom1 and drd1-6 for line 79). For both lines, however, we did successfully introgress the ddm1 mutation, which released silencing of the RP-FP fusion protein and reduced DNA methylation of the operator repeats in lines 101, 107, 112 (Fig. 2A, B). The bars indicate 2 mm. (A) Line 79 displayed strong derepression of the DsRed-LacI gene in a ddm1 mutant background (right). Line 26 was exceptional in that the EGFP-LacI gene was not strongly silenced in wild type plants and no improved expression was observed in the ddm1 mutant (left). (B) In both 79 and 26 lines, the operator repeats lost methylation. [file 1746-4811-6-2-S1.PDF]

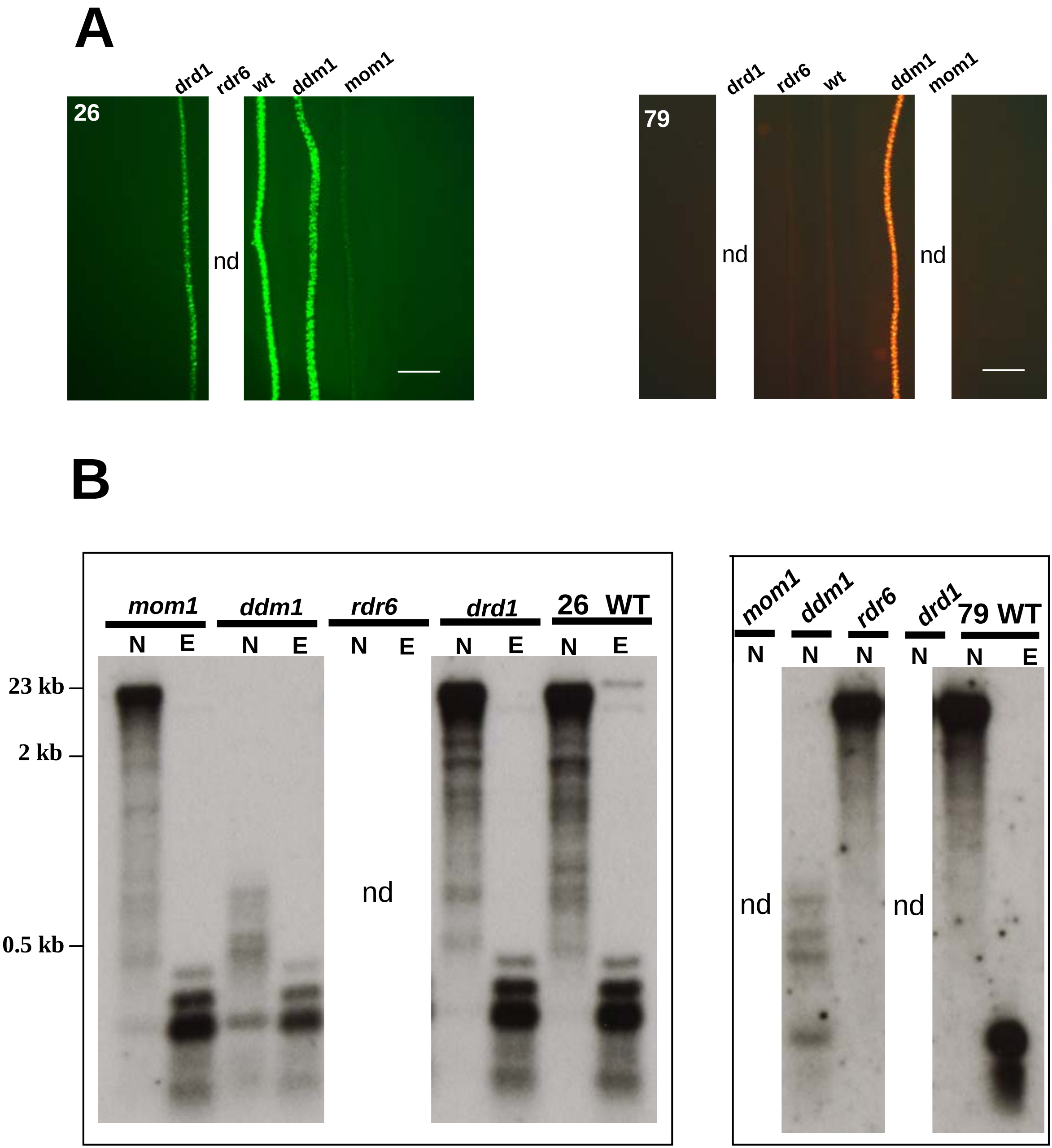
A
drd1
rdr6
wt
ddm1
mom1
26
nd
drd1
rdr6
wt
ddm1
mom1
79
nd
nd
B
mom1
ddm1
rdr6
drd1
26 WT
N E N E N E N E N E
23 kb
2 kb
0.5 kb
nd
mom1
ddm1
rdr6
drd1
79 WT
N N N N N E
nd
nd

Additional file 1: Release of silencing of the RP-FP fusion protein and loss of DNA methylation from *lac* operator repeats in a *ddm1* mutant.

For two lines, 26 and 79, we were not able to successfully introgress all four epigenetic mutations (*rdr6* for line 26, and for *mom1* and *drd1-6* for line 79). For both lines, however, we did successfully introgress the *ddm1* mutation, which released silencing of the RP-FP fusion protein and reduced DNA methylation of the operator repeats in lines 101, 107, 112 (Fig. 2A, B). The bars indicate 2 mm.

(A) Line 79 displayed strong derepression of the *DsRed-LacI* gene in a *ddm1* mutant background (right). Line 26 was exceptional in that the *EGFP-LacI* gene was not strongly silenced in wild type plants and no improved expression was observed in the *ddm1* mutant (left). (B) In both 79 and 26 lines, the operator repeats lost methylation.
