# Supplementary material for: High frequency, cell type-specific visualization of fluorescent-tagged genomic sites in interphase and mitotic cells of living Arabidopsis plants
Source: Plant Methods. 2010 Jan 19;6:2. doi: 10.1186/1746-4811-6-2 (PMC2820019; doi:10.1186/1746-4811-6-2)
Supplement: Additional file 2 — Tracking of fluorescent tagged sites on mitotic chromosomes. A 3D stack with 33 planes at intervals of 0.2 μm was taken over a period of approximately 4 minutes starting on a metaphase plate in homozygous line 107. Chromosome movement commenced at the beginning of the 4 minute period. Four still shots from a movie (Additional file 3: Movie Metamorph) are shown at the top. Left to right: plane 4, 10, 18, 24. The optical sections can be analyzed in ImarisTrack (Bitplane, Zürich) by changing the Z stack into a time stack and then the moving dots can be tracked and displayed in a rendered computer simulation (Additional file 4: Movie Imaris). Representative frames are shown at the bottom. [file 1746-4811-6-2-S2.PDF]

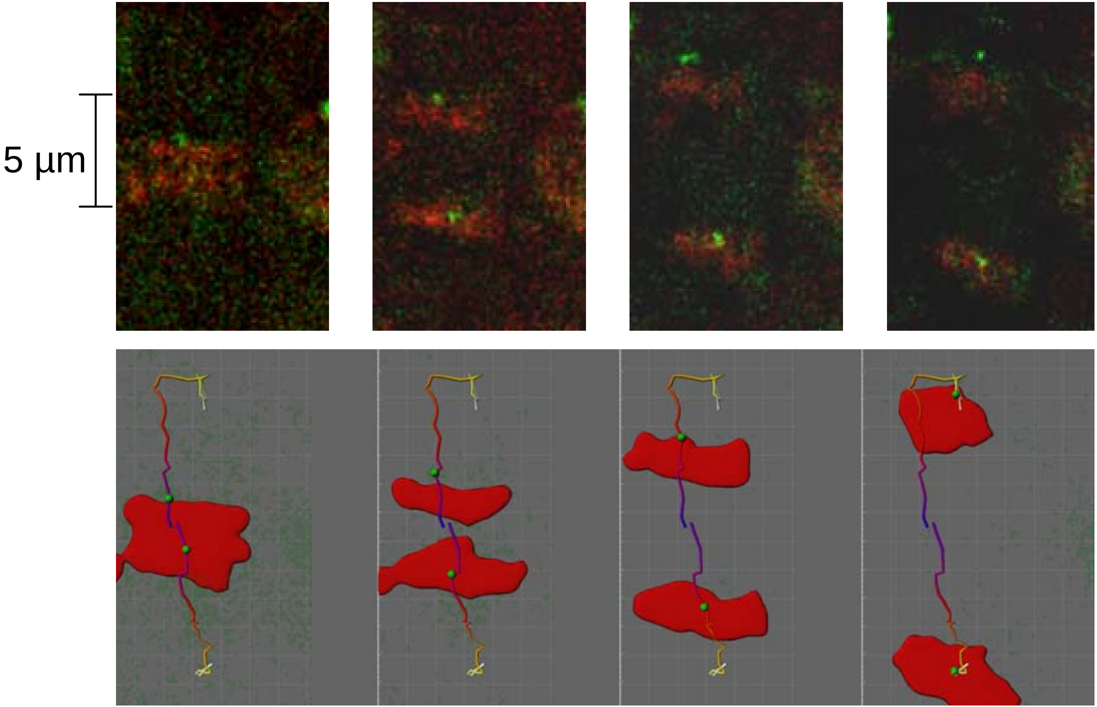
5 µm

Additional file 2: Tracking of fluorescent tagged sites on mitotic chromosomes. A 3D stack with 33 planes at intervals of 0.2 µm was taken over a period of approximately 4 minutes starting on a metaphase plate in homozygous line 107. Chromosome movement commenced at the beginning of the 4 minute period. Four still shots from a movie (Additional file 3: Movie Metamorph) are shown at the top. Left to right: plane 4, 10, 18, 24. The optical sections can be analyzed in ImarisTrack (Bitplane, Zürich) by changing the Z stack into a time stack and then the moving dots can be tracked and displayed in a rendered computer simulation (Additional file 4: Movie Imaris). Representative frames are shown at the bottom.
